# Permanganate of Potash in Gonorrhœa

**Published:** 1864-08

**Authors:** John G. Rich

**Affiliations:** Beachville, Canada West


### PERMANGANATE OF POTASH IN GONORRHŒA.

By JOHN G. RICH, M. D., Beachville, Canada West.

For the last two years I have frequently employed the permanganate of potash as an injection in the treatment of gonorrhœa, and the constant success derived from its use has been extremely satisfactory.

My usual method had previously been to administer, first, a hydragogue cathartic, then to give a mixture of cubebs, copaiva, nitre, &c., with injections of sulphate of zinc, tannic acid, &c. But since employing the permanganate my treatment has been much more circumscribed, for with this remedy alone, I have frequently cured very bad cases in forty-eight hours, and this too without its being followed by any evil effect from the sudden arrest of the discharge.

My usual mode of treatment, however, is as follows:—

℞ Potassæ Bitart. ℈j; Podophyllin, gr. j. M. In chartulas quatuor dividendus. S. One every two hours until free catharsis is produced.

After which:

℞ Potassæ Permangan, gr. vj; Aquæ Fontan, ℥j. M. S. To be used as an injection three times a day.

I direct at the same time the free employment of mucilaginous drinks, as althœa, ulmus, acacia, &c., and put the patient upon a non-stimulating regimen.

Out of sixty-four registered cases this course of treatment has failed in but two instances. And I find that recent attacks usually become arrested by it after from three to six injections. I have found it advisable to continue the demulcents for at least a week after the cessation of the discharge. In none of all these cases was the injection continued after the fourth day.

When accompanied by chordee, I usually employ the following:—

℞ Lupulin, ℈jss; Pulv. Camphoræ, ℈j; Micæ Panis, q. s. M. Ft. mass in pilulas, xvi, dividenda. S. Two, three, or four on going to bed.

I think that the permanganate of potash is a remedy deserving of more notice than physicians have hitherto given it, and hoping that my experience may produce for it a more extended trial in cases of gonorrhœa, I remain, &c.,

